# Wetting properties of poultry litter and derived hydrochar

**DOI:** 10.1371/journal.pone.0206299

**Published:** 2018-10-26

**Authors:** Vivian Mau, Gilboa Arye, Amit Gross

**Affiliations:** 1 Department of Environmental Hydrology and Microbiology, Zuckerberg Institute for Water Research, Ben Gurion University of the Negev, Campus Sde Boqer, Midreshet Ben Gurion, Israel; 2 French Associates Institute for Agriculture and Biotechnology of Drylands, Jacob Blaustein Institutes for Desert Research, Ben Gurion University of the Negev, Campus Sde Boqer, Midreshet Ben Gurion, Israel; RMIT University, AUSTRALIA

## Abstract

Detailed assessment of hydrochar wetting properties, which could provide an essential understanding of underlying mechanisms during its application to soils, is lacking. We characterized hydrochar produced from hydrothermal carbonization (HTC) performed on poultry litter at various temperatures and for different times in terms of hydrophobicity and surface free energy properties. Hydrochar was more hydrophobic than untreated poultry litter, and its hydrophobicity increased with increasing HTC temperature (contact angle > 130°). These changes were correlated with degradation of hemicellulose and cellulose. Hydrochar produced at 250°C contained mostly lignin and displayed high hydrophobicity over both prolonged wetting periods and repeated wetting cycles. Surface free energy was calculated using the Owens–Wendt–Rabel–Kaelble and Wu models, with the latter resulting in lower standard errors. The surface free energy decreased as HTC treatment severity increased from 26 mJ/m^2^ in the poultry litter to 8 mJ/m^2^ after treatment at 250°C for 60 min. The dispersive component fraction of the surface free energy increased with increasing treatment severity. This study demonstrated that changes in the physical composition of hydrochar due to increased treatment severity increase its hydrophobicity and decrease its surface free energy. Moreover, due to non-persistent hydrophobicity, hydrochar produced at temperatures lower than 250°C will likely not show adverse effects on soils.

## Introduction

Maintenance of soil fertility has been an issue in agriculture since its inception [1]. Continuous land cultivation leading to nutrient depletion, and soil erosion resulting in loss of soil organic matter are extant problems to this day [1,2]. To solve them, soil amendments such as animal manure and compost are commonly used. However, these can lead to pollution of surface and groundwater, and spread of pathogens, heavy metals, and pharmaceuticals [3]. Recently, these materials have begun to be dried and treated by pyrolysis to improve their properties and reduce the presence of pathogens [4]. Biochar, the product of pyrolysis, has been widely investigated in the last 20 years as a potential soil amendment, and has been found to improve soil fertility in some cases [5,6]. A meta-analysis on the effect of biochar application to soils on crop productivity determined that positive effects are most noticeable in soils with acidic to neutral pH and coarse to medium texture, at application rates of 100 t/ha [7]. Crop yield increase is mainly due to improved water-holding capacity and nutrient availability. Moreover, of the studied feedstocks, poultry litter demonstrated the greatest improvement in crop productivity [7].

Today, another possible treatment is being considered to produce soil amendments. Hydrothermal carbonization (HTC) is a fairly new technology that converts wet organic matter into a char-like compound termed hydrochar. Common feedstock for this technology are animal manures, sewage sludge and lignocellulosic agricultural waste [8–11]. Typically, HTC takes place in a temperature range of 180–250°C, under autogenous pressure, and reaction times varying from minutes to several hours [12]. Anoxic or low oxygen concentrations are required to retain the carbon in its solid form. Treating wet organic matter by HTC is intrinsically more energy efficient than other thermal processes, such as pyrolysis, because a specific phase change is avoided by heating at pressures greater than saturated pressure [13,14]. Therefore, it has been suggested for the treatment of animal manure, which has a naturally high moisture content. Traditionally, hydrochar is considered an energy source [14,15]; however, hydrochar has also been proposed as a potential soil amendment due to some similarities with the properties of biochar [8], but only a handful of studies have explored this possibility [10,16,17]. Moreover, a detailed comparison reveals many physico-chemical differences between the two products [15]. For example, hydrochar possesses an acidic pH, high N concentration, and aliphatic structure, whereas biochar has a basic pH, low N concentration, contains mostly aromatic structures, and has high thermal stability [9,18,19]. Consequently, the effect of hydrochar on soil properties needs to be thoroughly investigated.

A characterization of hydrochar wetting properties is essential to understanding the underlying mechanisms during its application to soil. Hydrochar has been found to be more hydrophobic than the raw material from which it is produced, based on analysis of functional groups on its surface, and the ease of separation of hydrochar from the liquid phase [11,12,20]. Some studies have indirectly assessed hydrochar hydrophobicity by measuring equilibrium moisture contents [21–24]. However, to the best of our knowledge, only two studies have directly investigated hydrochar hydrophobicity [16,20]. Results from those studies suggested that temperature and time of digestion, or in other words, the severity of the HTC, affect the hydrophobic properties of the hydrochar, although the effect may be substrate-dependent. For example, an increase in temperature from 200 to 250°C resulted in increased hydrophobicity for hydrochar produced from corn digestate but not for hydrochar produced from woodchips [16]. Knowledge of hydrochar wetting properties is still limited and warrants special consideration.

This study provides a detailed assessment of the wetting properties of hydrochar produced from poultry litter. Poultry litter, a mix of poultry manure, bedding materials, feathers, and spilled feed, was chosen because its direct application to fields can result in spread of pathogens, and contamination of water bodies [25], and stringent regulations such as the European Union nitrates directive result in the need to treat it [26]. Moreover, produced quantities are on the rise as poultry production is growing at a global annual rate of 1.6% [27], generating 625–938 million tons of litter [28,29]. The natural moisture content of poultry litter indicates that treatment by HTC would be more energetically efficient than pyrolysis [14]. Finally, positive experiences with application of poultry litter-derived biochar to soils [7,25] indicates that this feedstock has potential as a soil amendment. By characterizing hydrochar wetting properties through hydrophobicity and surface free energy components, it is possible to elucidate its behavior as a potential soil amendment. Specifically, this study had three main goals: (i) to characterize the effect of HTC treatment temperature and time on poultry litter-derived hydrochar hydrophobicity; (ii) to investigate the persistence of its hydrophobicity during wetting cycles; and (iii) to determine the surface free energy components of the hydrochar that govern the hydrophobic effects.

## Materials and methods

### Hydrochar production

Hydrochar was produced from poultry litter originating from a chicken farm in the Negev region of Israel following the procedure outlined by Mau et al. [30]. Briefly, the poultry litter consisted of chicken feces, wood chips, spilled feed, and feathers. Clean wood chips were placed in the house when a new flock of chicks was introduced. When the chickens achieved maturity at 22 weeks, the farm staff removed the poultry litter from the house. With permission from the farm owner, the researchers obtained the poultry litter for experiments in the laboratory. It was then dried at 105°C for 24 h, and aggregates were crushed using a mortar and pestle and then sieved through a no. 8 mesh. The dried and homogenized feedstock was stored in a desiccator prior to HTC experiments. The dried poultry litter was mixed with doubled-distilled water at a solid-to-water ratio of 1:3. The poultry litter sludge was placed in HTC reactors, one of which had a temperature probe to provide a representative measure of the temperature inside the reactors. Reactors were heated by immersion in Paratherm HR heat-transfer fluid (Conshohocken, PA) that was preheated to temperatures of 180°C, 220°C and 250°C. Once the reactors reached the set temperatures, carbonization was run for 5, 30 and 60 min. When the desired reaction time had elapsed, the reaction was quenched by placing the reactors in an ice bath. All combinations of temperature and reaction time were conducted in triplicate using the same poultry litter to ensure repeatability. All subsequent analyses were performed separately for each sample or replicate. The generated hydrochar was separated from the liquid phase and oven-dried at 105°C for 24 h. The dried hydrochar was ground using a mortar and pestle and then sieved. The <150 μm fraction was used in the study.

Fiber analysis was conducted to determine the poultry litter and hydrochar contents of hemicellulose, cellulose and lignin by the van Soest method of neutral detergent fiber, acid detergent fiber and acid detergent liquid (aNDF–ADF–ADL) [31]. This was performed with a fiber analyzer (model A200, Ankom Technology, Macedon, NY) following the manufacturer's procedures [32–34] at the Forage and Feed laboratory (Gedera, Israel). Due to the need for a large amount of sample for these analyses, they were performed in duplicates from a composite sample formed by mixing the triplicate samples of each treatment.

### Contact-angle measurements

Contact angle (CA), a measure of hydrophobicity, was estimated by the sessile drop method and the Wilhelmy plate method. The first method measured the stability of hydrochar wettability over time [35], and the initial CA was used for the calculation of surface free energy components. The Wilhelmy plate method [36] was used to estimate the advancing and receding dynamic CA under multiple wetting cycles, thus assessing the persistence of hydrophobicity. Applying both methods provides a more complete evaluation of the hydrophobic properties of the material and its behavior over time.

#### Sessile drop method

Hydrochar hydrophobicity was assessed using the sessile drop method according to Bachmann et al. [37]. Specifically, the hydrochar particles were sprinkled on double-sided adhesive tape placed on a glass slide. The excess particles on the slide were removed by gentle tapping until a one-grain-thick hydrochar layer was obtained. The CA formed between the hydrochar and a water droplet was monitored as a function of time. A 10-μL droplet of double-distilled water was placed on the hydrochar-coated slide and the CA was measured over a period of 3 min at 30 frames per second using an optical goniometer (OCA20, Dataphysics, Filderstadt, Germany). The horizontal view of the water drop on the hydrochar slide was used to calculate the CA with the software SCA 20 (Dataphysics). The CA was measured for hydrochar produced at all temperatures and reaction times, as well as for the untreated poultry litter. In a similar manner, the initial advancing CAs of four additional wetting liquids (ethylene glycol, formamide, glycerol and diiodomethane) were measured. The initial advancing CAs for all wetting liquids were used to calculate the surface free energy components as detailed in the section on surface free energy calculations.

#### Wilhelmy plate method

The dynamic CA was evaluated by the Wilhelmy plate method as described by Bachmann et al. [36] with slight modifications [38]. Specifically, glass slides (76 mm long, 26 mm wide and 1 mm thick) were covered with a double-sided adhesive tape. Each slide was sprinkled with hydrochar particles until the tape on both sides and edges was completely concealed [36]. Excess particles were removed until a one-grain-thick hydrochar layer was obtained. Slides were secured (one at a time) to an electronic micro balance (DCAT 11, Dataphysics) with surfaces perpendicular to a glass vessel containing double-distilled water. Slides were immersed in the glass vessel to a depth of 5 mm at a rate of 0.2 mm/s and subsequently emersed at the same rate. Four immersion/emersion cycles were performed with 1-s pause between cycles. The immersion/emersion process and the corresponding CA calculations were performed with SCAT-12 software (Dataphysics).

The advancing and receding CAs were calculated from the total force (Ft, kg) measurements during immersion and emersion, respectively, according to Eq 1.

$$cos\;\theta =\frac{\left(F_{t}+V\rho g\right)}{l_{w}\gamma_{lv}}$$ (1)

where *θ* (°) is the CA, *V* (m^3^) is the volume of the immersed section of the slide, *ρ* (kg/m^3^) is the density of the wetting liquid, *g* (m/s^2^) is the acceleration due to gravity, l_*w*_ (m) is the wetted perimeter and *γ*_*lv*_ (N/m) is the surface tension of the wetting liquid at the liquid/vapor interface. For each cycle, the CA hysteresis (i.e., the difference between the advancing and receding CAs) was calculated, serving as an additional measure for hydrophobicity persistence.

Three measurements were performed for each hydrochar replicate produced at 180°C, 220°C and 250°C at a reaction time of 60 min, and the poultry litter. Thus, in total, 9 measurements were performed for each treatment temperature and the untreated material.

### Surface free energy calculations

Additional quantitative insights into the surface characteristics of the poultry litter and derived hydrochars can be gained from the surface free energy (mJ/m^2^) at the solid/air interface, which can also be viewed as the interfacial tension (mN/m). Following the concept that interfacial tensions (solid/air or liquid/air) are comprised of additive polar and dispersive (i.e., apolar) components, one can calculate the interfacial tension at the solid/air interface from knowledge of interfacial tension of the wetting liquids and their corresponding CA. As mentioned, the sessile drop method was used to measure the CA formed by ethylene glycol, formamide, glycerol and diiodomethane on the surface of the poultry litter and derived hydrochar. The surface tension of the wetting liquids and their polar and dispersive components are shown in Table 1.

**Table 1:** Surface free energy components of the wetting liquids.

Liquid	Surface free energy (mJ/m^2^)		
	γ_l_	γ_l_^d^	γ_l_^p^
Water	72.8	22.1	50.7
Ethylene glycol	47.7	26.3	21.4
Formamide	58.2	39.0	19.2
Glycerol	63.4	29.0	34.4
Diiodomethane	50.8	50.8	0

The consistency between two models that quantify the solid surface free energy and its components were examined: (i) the Owens–Wendt–Rabel–Kaelble (OWRK) method [39,40] (Eq 2) and (ii) the Wu method [41] (Eq 3).

$$\gamma_{l}(1+\cos\;\theta )=2\;\sqrt{\gamma_{s}^{d}\gamma_{l}^{d}}+2\sqrt{\gamma_{s}^{p}\gamma_{l}^{p}}$$ (2)

$$\gamma_{l}(1+\cos\;\theta )=4\;\left(\frac{\gamma_{s}^{d}\gamma_{l}^{d}}{\gamma_{s}^{d}+\gamma_{l}^{d}}+\frac{\gamma_{s}^{p}\gamma_{l}^{p}}{\gamma_{s}^{p}+\gamma_{l}^{p}}\right)$$ (3)

where **γ** (mJ/m^2^) is the interfacial tension; θ (°) is the measured CA, the subscripts *l* and *s* stand for the liquid and solid phases, respectively, and the superscripts *d* and *p* stand for the dispersive and polar components, respectively. The surface free energy calculations were carried out using the SCA 21 software (Dataphysics).

### Statistical analysis

Analysis of variance was conducted to determine statistical differences between treatments (*p* < 0.05). Treatment temperature and time were set as the fixed factors, and the above-described measured or calculated parameters were the dependent variables. When a significant difference was detected, Tukey’s post hoc test was performed. For surface free energy experiments, an exponential decay regression analysis was performed. The severity factor was set as the independent variable, and the surface free energy was set as the dependent variable. Analysis of variance analyses were performed with Statistica version 10 (StatSoft, Tulsa, OK), and regression analyses were performed with SigmaPlot 12.5 (Systat Software Inc., Chicago, IL).

## Results and discussion

**Fig 1:**
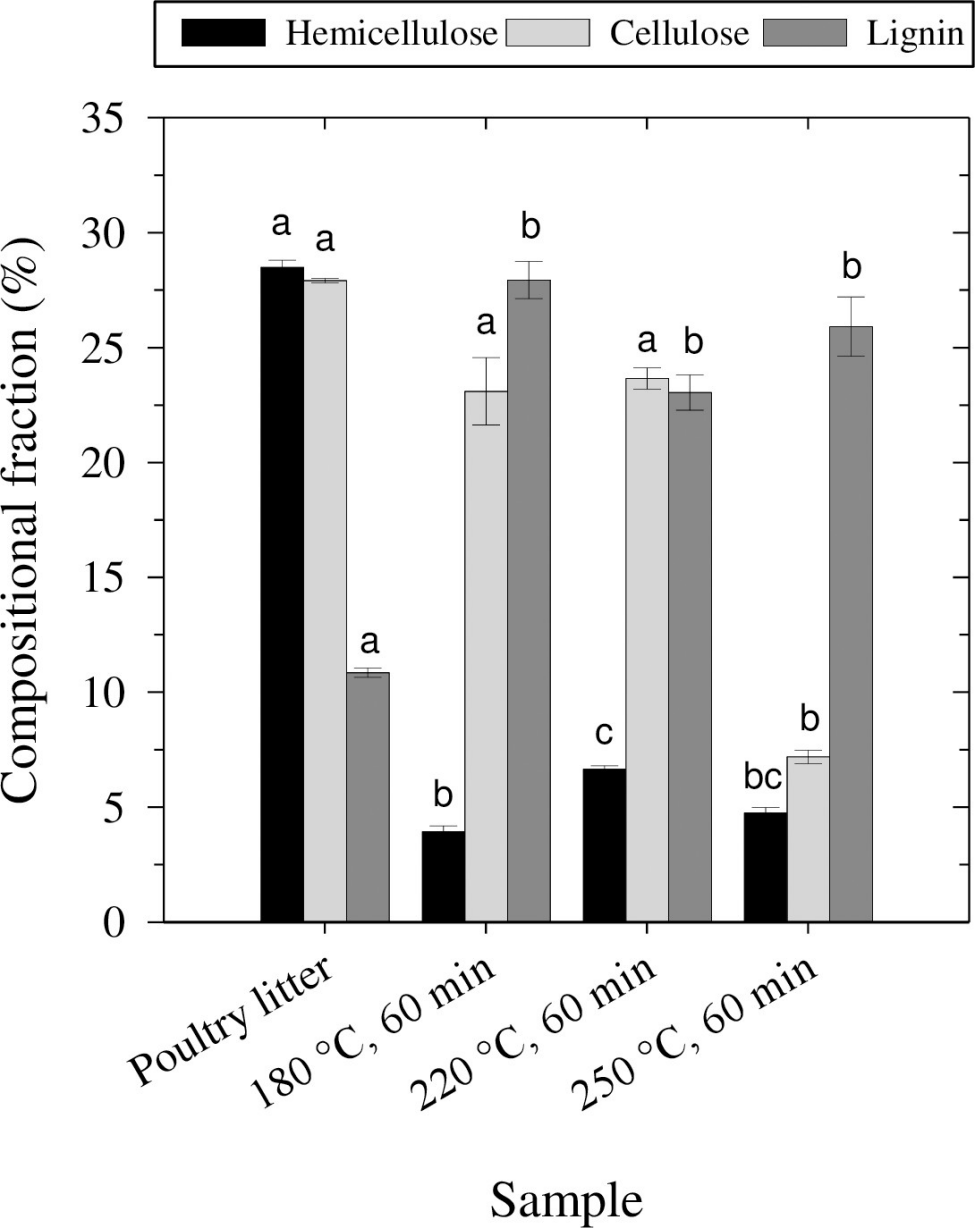
Fiber composition of poultry manure and hydrochar. Data for hydrochar produced at 180°C, 220°C and 250°C after 60 min of treatment is shown. Lowercase letters indicate significant differences between samples for each fiber.

The poultry litter and derived hydrochar were characterized in terms of fiber composition, specifically hemicellulose, cellulose and lignin fractions. The hemicellulose fraction decreased significantly (*p* < 0.05) from 28% in the poultry litter to less than 7% in hydrochar produced at all temperatures after 60 min (Fig 1). The HTC process degraded hemicellulose, a hydrophilic fiber, resulting in a hydrochar composed of more hydrophobic fibers—cellulose and lignin [15]. At the treatment temperature of 250°C, significant cellulose degradation was observed (*p* < 0.05). The resulting hydrochar was mainly composed of lignin fibers, differentiating it from those produced at 180 and 220°C. In total, the fibers accounted for 38–67% of the hydrochar composition. The remaining 33–62% consisted of ash, sugars, protein, fat and starch. For example, at 250°C, the cellulose fraction underwent degradation, probably forming sugars—which were not measured, thus reducing the fraction of the hydrochar composition that was accounted for. A general chemical characterization of the poultry litter and hydrochars focusing on elemental composition and FTIR spectra can be found in Mau et al. [30].

### Time-dependent contact angle

**Fig 2:**
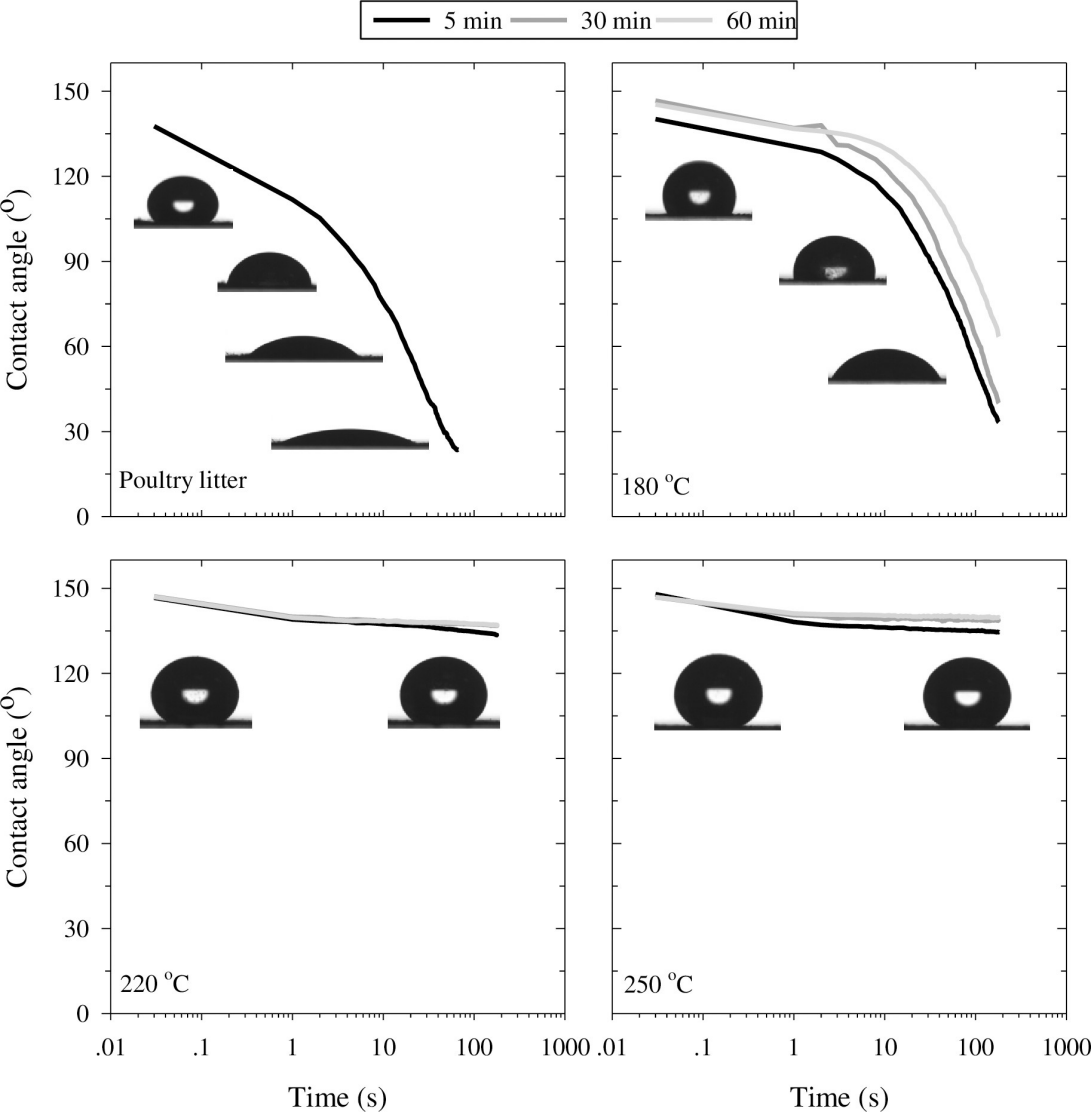
Contact angle of water drop formed on the surface of poultry litter and derived hydrochar. Data for hydrochar generated at 180°C, 220°C, and 250°C at 5, 30 and 60 min is shown. Standard errors were less than 2% for all curves, except for poultry litter and hydrochar produced at 180°C after 5 and 30 min, where it was up to 13%. Photos demonstrate drops created on samples after 60 min of carbonization.

The time-dependent CA of a water drop placed on the surface of poultry litter before and after the HTC treatments (Fig 2) served as a quantitative measure for the degree of hydrophobicity and its persistence. For each surface, the initial advancing CA was calculated from the first frame (corresponding to about 33 ms). The average value obtained from all hydrochar samples was 145° ± 3° with no significant difference between HTC production temperature and time (*p* > 0.15). The initial CA obtained for the poultry litter was 138° and found to be significantly different (*p* < 0.05) from all hydrochar samples.

For all samples, a rapid decrease in CA was observed in the first few seconds of measurement (Fig 2). This rapid decrease continued for the untreated poultry litter, and at a slower rate for samples generated at 180°C. After 67 s, the CA for poultry litter decreased to 22° and could no longer be determined with precision. After 3 min of measurement, the CA decreased to 33°, 40°, 63° for the 180°C hydrochar produced after 5, 30 and 60 min, respectively (Fig 2). With respect to the hydrochars produced at temperatures of 220 and 250°C, after the initial rapid decrease, the CAs remained elevated, relatively constant and statistically similar to each other. In total, after 3 min, the reduction in CA was of 13° or less, remaining above 130° (Fig 2). These results are similar to sessile drop method experiments performed on woodchip-derived hydrochar that was carbonized at 200 and 250°C for 6 h [16]. The woodchip hydrochar was also highly hydrophobic (initial CA > 130°), and over 5 s, the CA decreased by about 5 and 15° for hydrochar produced at 250 and 200°C, respectively.

The marked difference between the degree of hydrophobicity of untreated poultry litter and hydrochar can be explained in part by the fiber composition of the materials. The poultry litter consisted of 28% hemicellulose, a hydrophilic fiber, which was degraded to less than 7% during HTC.

It should be noted that in the sessile drop method, the sample's roughness can influence CA measurements, leading to a higher CA than would be measured on a smooth surface [37]. This might explain in part why the CAs measured in this study were so much higher than those measured by He et al. [20] on compressed disks of sewage-sludge-derived hydrochar.

### Surface free energy components

As already noted, the initial advancing CAs from the sessile drop method for poultry litter and hydrochars were similar. Since water is a highly hydrophilic wetting liquid (72 mJ/m^2^) and the hydrochars were found to be highly hydrophobic, it was difficult to distinguish between the surface characteristics of the different hydrochars from the initial advancing CA, when water was the wetting liquid. Therefore, further CA measurements were conducted with the sessile drop method using other wetting liquids with lower total surface tension and different polar and dispersive components (Table 1).

To combine the effects of varying HTC temperatures and reaction times, we used the severity factor (SF), developed by modeling HTC kinetics according to hydrochar oxygen loss [42]. The model was calibrated with data from HTC of feedstocks ranging from cellulose to sub-bituminous coal treated at temperatures of 120–390°C and reaction times ranging from 1 min to 6 months.

$$SF={50*t}^{0.2}*exp(-3500/T)$$ (4)

where t is the reaction time in seconds, and T is the temperature in K. According to the SF, different combinations of time and temperature that lead to the same SF may result in similar effects on reactions and hydrochar properties. The SFs of the studied poultry litter and derived hydrochars are presented in Table 2.

**Table 2:** Polar (γ_p_) and dispersive (γ_d_) surface free energy components calculated using OWRK and Wu models for hydrochar generated at various reaction temperatures and times. The effect of temperature and time is combined in the severity factor (SF; [42]). Values are presented as mean ± standard error. In parentheses is the fraction of total surface free energy.

Sample	SF	OWRK		Wu	
		γ_p_	γ_d_	γ_p_	γ_d_
		(mJ/m^2^)		(mJ/m^2^)	
Poultry litter	0.00	28.8 ± 7.0	1.5 ± 0.9 (0.0 ± 0.0)	25.9 ± 5.0	0.2 ± 0.2 (0.0 ± 0.0)
180°C, 5 min	0.07	5.3 ± 3.8	13.6 ± 7.5 (0.6 ± 0.2)	7.8 ± 3.2	10.6 ± 4.5 (0.5 ± 0.2)
180°C, 30 min	0.10	4.1 ± 2.8	8.0 ± 3.7 (0.6 ± 0.3)	7.8 ± 4.0	10.5 ± 6.0 (0.5 ± 0.2)
180°C, 60 min	0.11	2.0 ± 0.7	5.0 ± 1.5 (0.7 ± 0.1)	6.3 ± 1.1	5.3 ± 1.5 (0.4 ± 0.1)
220°C, 5 min	0.13	1.2 ± 0.4	11.9 ± 4.3 (0.8 ± 0.1)	2.1 ± 1.2	9.3 ± 2.7 (0.8 ± 0.1)
220°C, 30 min	0.18	1.0 ± 0.4	11.6 ± 2.0 (0.9 ± 0.0)	1.3 ± 0.9	7.6 ± 0.7 (0.9 ± 0.1)
220°C, 60 min	0.19	1.9 ± 0.3	10.9 ± 3.7 (0.8 ± 0.1)	2.7 ± 2.2	9.0 ± 2.9 (0.7 ± 0.2)
250°C, 5 min	0.21	0.7 ± 0.3	11.0 ± 1.8 (1.0 ± 0.0)	0.8 ± 0.6	9.4 ± 1.4 (0.9 ± 0.1)
250°C, 30 min	0.28	0.3 ± 0.2	7.0 ± 0.9 (1.0 ± 0.0)	1.0 ± 0.5	7.0 ± 0.8 (0.9 ± 0.1)
250°C, 60 min	0.32	0.9 ± 0.3	3.0 ± 1.5 (0.6 ± 0.2)	5.7 ± 2.6	2.8 ± 1.4 (0.4 ± 0.2)

**Fig 3:**
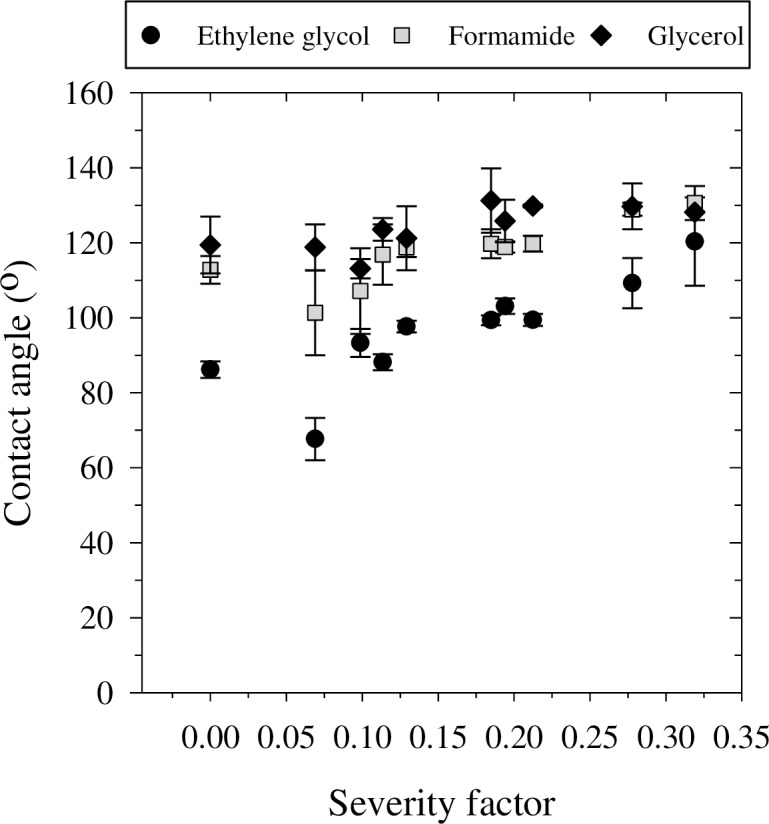
Contact angles measured for poultry litter and hydrochar. Contact angles were measured using different test liquids, poultry litter, and hydrochar generated at 180°C, 220°C, and 250°C at 5, 30 and 60 min. The effect of temperature and time is combined in the severity factor [42]. Bars indicate standard errors.

In Fig 3, the CA as a function of SF is presented for the wetting liquids employed (Table 1), excluding diiodomethane. The latter exhibited instantaneous and complete wetting (i.e., CA = 0) when placed on any one of the surfaces. It should be noted that the total surface tension of diiodomethane is moderate (50.8 mJ/m^2^), but its polar fraction is zero. Namely, wetting interaction took place between the dispersive fraction of the liquid and solid phases, implying a highly hydrophobic nature for the hydrochar.

In contrast to the similar CAs obtained for water, the CAs obtained for ethylene glycol, formamide and glycerol were sensitive to the HTC treatments. Specifically, for these wetting liquids, the CA as a function of SF (Fig 3) exhibited a sigmodal-like pattern of increasing CA with increasing SF. In general, the CA values corresponded to the total surface tension, where the CA obtained for glycerol (63.4 mJ/m^2^) was highest and that obtained for ethylene glycol (47.7 mJ/m^2^) was lowest. At the highest SF (0.32), however, no significant differences could be observed among the three wetting liquids.

**Fig 4:**
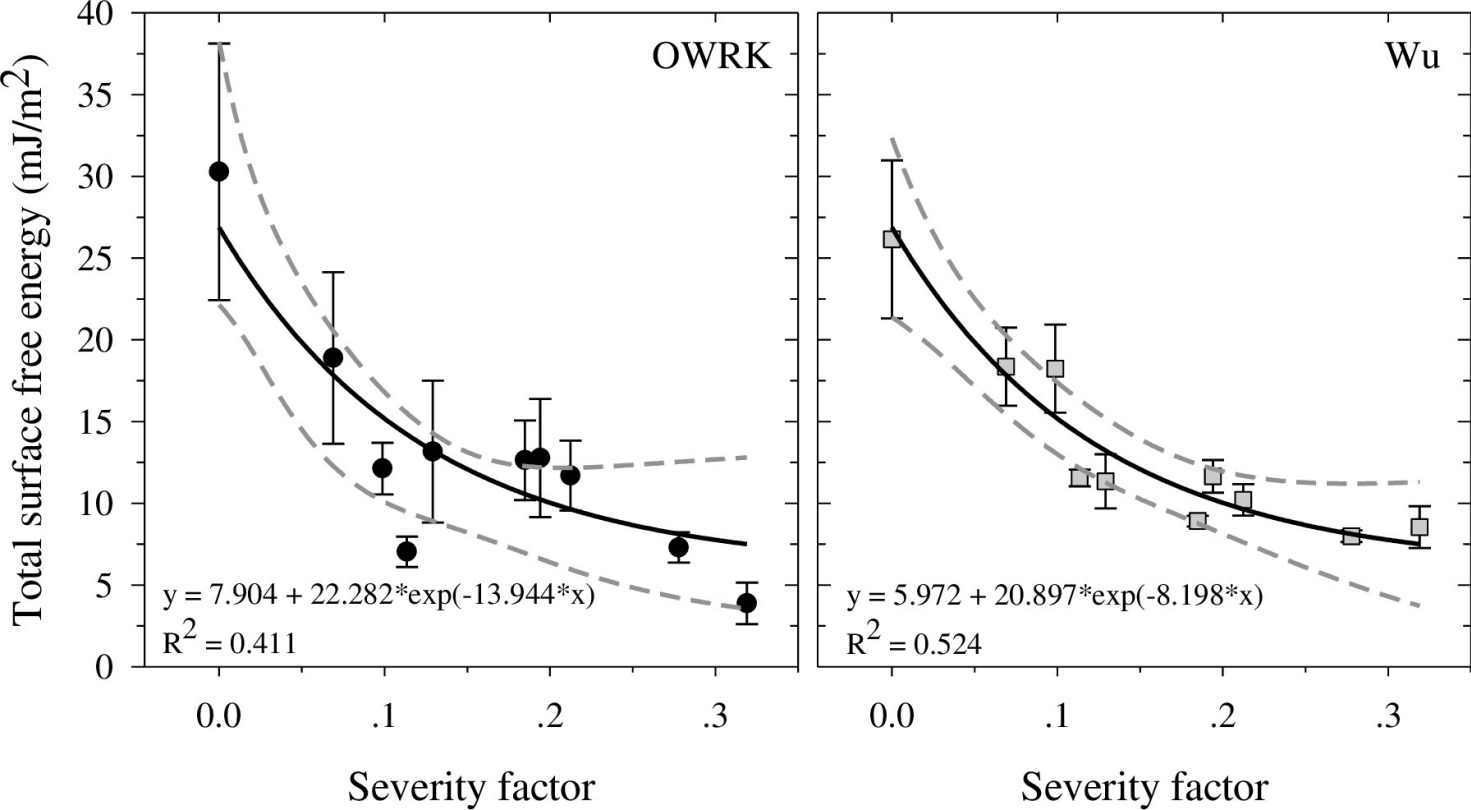
Total surface free energy of hydrochar calculated using OWRK and Wu models. Calculation was performed with values from poultry litter and hydrochar generated at 180°C, 220°C, and 250°C at 5, 30 and 60 min. The effects of temperature and time are combined in the severity factor [42]. Bars indicate standard errors. Solid lines indicate the regression line, and the dotted lines the 95% confidence bands. Regression equation and coefficient of determination are shown for each model.

The measured CA values (Fig 3) were further used to calculate the surface free energy and its components using the Wu and OWRK models. The resultant total surface free energies as a function of SF are presented in Fig 4, together with regression curves and 95% confidence bands. Both models produced total surface free energy values of similar magnitude, which followed a similar trend. The Wu model resulted in smaller standard errors than the OWRK model, especially for samples with SF > 0.13. As the SF increased, the surface free energy decreased following an exponential decay. The Wu model demonstrated a better correlation with the SF (Fig 4). The total surface free energy of the untreated poultry litter (i.e., SF = 0) was about 26–30 mJ/m^2^, whereas the surface free energy for hydrochar with SF ≥ 0.11 was about 10 mJ/m^2^. These values are relatively low, implying that the hydrochar surface is extremely hydrophobic and may indirectly impact crop yields by inducing water repellency when added to mineral soils. In comparison, agricultural cultivated loess soils have a surface free energy of 39–68 mJ/m^2^ [43,44]. These high surface free energy values demonstrate the hydrophilic nature of the mineral soils in comparison to the hydrochar. Therefore, the addition of hydrochar to mineral soils could significantly change its hydrophobicity. Nevertheless, more research is necessary to assess the impact of hydrochar on soil's hydraulic properties in conjunction with crop yields. It should be noted, however, that the hydrophobicity of hydrochar produced at temperatures lower than 250°C was not persistent, as will be shown in the following section.

The OWRK and Wu models separate the surface free energy into dispersive and polar components. The two components and the fraction of the total surface free energy associated with the dispersive component are shown in Table 2. In general, the models agreed on the proportion of surface free energy associated with the dispersive component. This represented a close to negligible fraction of the surface free energy for the poultry litter. As the SF increased, the dispersive component increased and accounted for almost all of the total surface free energy, except for the hydrochar produced at 250°C after 60 min. In comparison, in agricultural cultivated loess soils, the dispersive component represents one-third of the surface free energy [43].

### Advancing and receding contact angles

**Fig 5:**
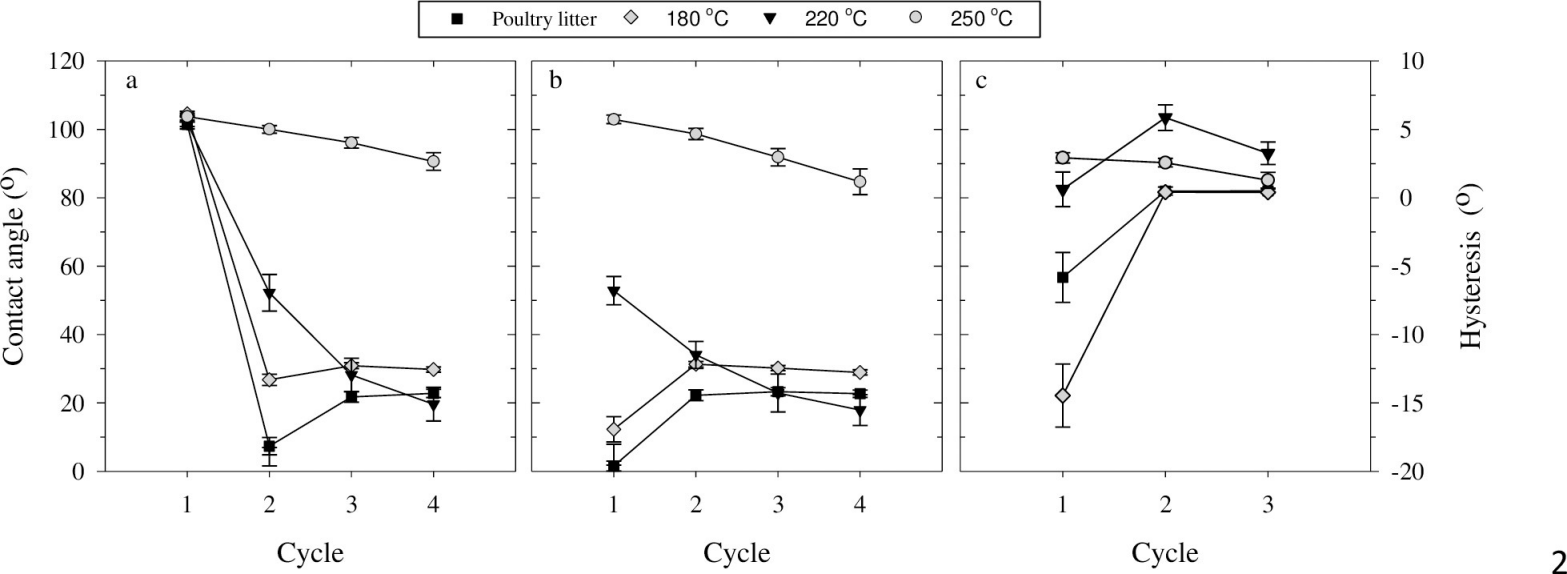
Contact angles measured with the Wilhelmy plate method. (a) Advancing and (b) receding contact angles, and (c) hysteresis for poultry litter and hydrochar generated at 180°C, 220°C, and 250°C after 60 min of treatment. Bars indicate standard error.

The advancing CA measured during the first cycle was similar for all samples (Fig 5A). This is in agreement with the similar initial advancing CAs obtained by the sessile drop method (Fig 2). However, the values obtained were smaller than the initial advancing CA measured with the sessile drop method. This disparity has also been observed in other studies [37,45] where it was found that in general, the Wilhelmy plate method underestimates the advancing CA because the actual wetted perimeter is larger than the perimeter of the slide due to the roughness created by the sample particles. Both methods, however, will yield higher advancing CAs relative to an ideal smooth surface with the same chemical characteristics. In general, the CA measured on rough hydrophobic surfaces (i.e., CA > 90°) is larger than it would be on an ideal smooth surface [37].

With subsequent wetting cycles, the advancing CA decreased drastically, by more than 74°, for the poultry litter and hydrochar produced at 180 and 220°C. The advancing CA of hydrochar produced at 250°C decreased only slightly with each wetting cycle; the advancing CA measured in cycle 4 was only 13° lower than that from the first cycle. The behavior observed for the 250°C hydrochar was similar to the effect of the prolonged static contact time investigated by the sessile drop method. In fact, the time elapsed to measurement of the advancing CA in cycle 4 was similar to the prolonged static contact time (153 vs. 180 s). Therefore, hydrochar produced at 250°C is highly hydrophobic over a long wetting period as well as over repeated wetting periods. On the other hand, hydrochar produced at 220°C is hydrophobic over long periods but not with subsequent wetting cycles.

The receding CA was measured when the slide was emersed from the water. The receding CA measured in cycle 1 was significantly smaller (*p* < 0.05) than the advancing CA of that same cycle for all samples excluding the 250°C hydrochar (Fig 5B). In other words, in cycle 1, hysteresis was high for poultry litter and hydrochar produced at 180 and 220°C, and close to zero for hydrochar produced at 250°C (Fig 5C). With each wetting cycle, hysteresis decreased for all samples, except for the 250°C hydrochar where hysteresis remained relatively constant and low throughout all cycles. The increase in receding CA observed from cycle 1 and 2 for poultry litter and hydrochar produced at 180°C was likely due to the methodology's lower sensitivity at low CA values. With each subsequent cycle, the receding CA converged to values similar to the advancing CA and thus, hysteresis was negligible.

The significant decrease in receding CA compared to advancing CA in cycle 1 for poultry litter and hydrochar produced at 180 and 220°C may be the result of water adsorption to the particles’ surface during measurement, whereas the surface was dry for the advancing CA measurement. Thereafter, the advancing CA measured in cycle 2 was similar to the receding CA measured in cycle 1 because it contained a similar amount of adsorbed water. It is well known that ordination and/or configuration of hydrophilic and hydrophobic functional groups at the surface is dependent upon their interaction with water. Specifically, when water is adsorbed, polar functional groups interact with the water molecules. However, as the surface dries, polar groups interact with each other, increasing the fraction of hydrophobic functional groups at the surface [46–48]. This mechanism might explain the rapid decrease in advancing CA and receding CA observed for poultry litter and hydrochar produced at 180 and 220°C.

The measurements of advancing and receding CA showed that hydrochar hydrophobicity is persistent for hydrochar produced at 250°C, but not for hydrochar produced at lower temperatures. This discernible difference can be explained by the hydrochars' chemical composition. The 250°C hydrochar has a lower amount of cellulose than the other hydrochars (Fig 1). Cellulose has both hydrophobic and hydrophilic components, and can reorient itself at the surface, resulting in more hydrophilic behavior [49]. Since the cellulose was degraded, the more hydrophobic lignin, which has less OH-binding sites for water [50], probably had a stronger influence on the hydrochar's hydrophobicity. A smaller presence of OH-bonds has been reported for this 250°C hydrochar [30]. Principal component analysis of FTIR spectra of these hydrochars also confirmed that the 250°C hydrochar is markedly different from the other hydrochars [30]. The persistent hydrophobicity of 250°C hydrochar implies that it could have adverse effects on soil hydraulic properties; however, hydrochar produced at lower temperatures could be a good soil amendment. More research is needed to determine hydrochar performance in supporting crop yield.

## Conclusions

Wetting properties of poultry litter and derived hydrochar were investigated. A hydrophobic material was generated by HTC, with treatment temperature having a greater influence than time. Only hydrochar produced at 250°C demonstrated hydrophobic behavior under repeated wetting cycles, probably due to lower amounts of cellulose which resulted in less water adsorption and hydrophilic molecule reorientation at the surface. Due to this persistent hydrophobicity, the hydrochar produced at 250°C could negatively impact soil hydraulic properties; however, based on its wetting behavior, hydrochar produced at lower temperatures would likely not present a problem when added to soil. The total surface free energy decreased with treatment severity. Poultry litter had a weak dispersive component, which increased with increasing severity of HTC treatment. Further experiments should be conducted to correlate hydrochar wetting characteristics with soil hydraulic properties, and subsequently with crop yield. This study contributes basic knowledge toward utilizing hydrochar as a soil amendment, thereby increasing its applications and value.
